# The effect of anxiety on cognition in older adult inpatients with depression: results from a multicenter observational study

**DOI:** 10.1016/j.heliyon.2019.e02235

**Published:** 2019-08-28

**Authors:** Liva Jenny Martinussen, Jūratė Šaltytė Benth, Ina Selseth Almdahl, Tom Borza, Geir Selbæk, Bodil Mcpherson, Maria Stylianou Korsnes

**Affiliations:** a Department of Old Age Psychiatry, Oslo University Hospital, Norway; b Institute of Clinical Medicine, Campus Ahus, University of Oslo, Norway; c Health Services Research Unit, Akershus University Hospital, Norway; d Institute of Clinical Medicine, University of Oslo, Norway; e Research Centre for Age-related Functional Decline and Disease, Innlandet Hospital Trust, Norway; f Norwegian National Advisory Unit on Ageing and Health, Vestfold Hospital Trust, Norway; g Department of Geriatric Medicine, Oslo University Hospital, Norway; h Department of Psychology, University of Oslo, Norway

**Keywords:** Psychiatry, Clinical psychology, Anxiety, Late-life depression, Cognition, Cognitive function, Older adults, Seniors

## Abstract

Late-life depression is associated with reduced cognitive function beyond normal age-related cognitive deficits. As comorbid anxiety frequently occur in late-life depression, this study aimed to examine the association between anxiety symptoms and cognitive function among older inpatients treated for depression. We hypothesized that there would be an overall additive effect of comorbid anxiety symptoms on dysfunction across cognitive domains. The study included 142 patients treated for late-life depression in hospital, enrolled in the Prognosis of Depression in the Elderly study. Anxiety symptoms were measured at admission using the anxiety subscale of the Hospital Anxiety and Depression Scale. Patients completed cognitive tasks at admission and discharge. Linear mixed and generalized linear mixed models were estimated to investigate the effect of anxiety, on continuous and categorical cognitive scores, respectively, while controlling for depression. Anxiety severity at admission was not associated with performance in any of the cognitive domains. Patients with more symptoms of anxiety at admission demonstrated a significant improvement in immediate recall during the hospital stay. Patients with a score above cutoff indicating clinically significant symptoms on the anxiety subscale performed better on general cognitive function, as measured by the Mini Mental Status Examination at admission, than those below cutoff for anxiety. In conclusion, comorbid anxiety symptoms had no additive effect on cognitive dysfunction in late-life depression in our sample of inpatients.

## 1 Introduction

Depression is commonly accompanied by anxiety (Löwe et al., 2008), especially in older adults (Lenze et al., 2000). Severe anxiety symptoms corresponding to a diagnosis of Generalized Anxiety Disorder (GAD) has been reported in nearly 60% of older inpatients diagnosed with depression (Bendixen and Engedal, 2016). Comorbid anxiety symptoms in late-life depression (LLD) has been associated with more severe depression (Bendixen et al., 2018; Lenze et al., 2000), worse treatment response (Andreescu et al., 2007) and higher suicidality (Bendixen et al., 2018), than LLD without comorbid anxiety.

Knowledge about if and how comorbid anxiety symptoms might affect cognitive functioning in LLD is more limited. LLD itself is associated with reduced cognitive function beyond normal age-related cognitive deficits (Koenig et al., 2014; Morimoto and Alexopoulos, 2013), and may be an independent risk factor or a prodromal stage of dementia (Diniz et al., 2013). The association between anxiety disorders and dementia is still uncertain (de Bruijn et al., 2014; Gulpers et al., 2016).

Compared to depression without co-occurring anxiety, comorbid anxiety disorders in LLD might be associated with a more severe decline in some cognitive domains relative to others. In one study, older adults with depression and a comorbid anxiety disorder had a greater decline in memory during a 4-year period compared to depressed older adults without a comorbid anxiety disorder. Other cognitive domains were however not affected (DeLuca et al., 2005). To our knowledge, only one research group has looked specifically at how anxiety symptoms in LLD influence cognitive function. Bendixen and colleagues studied older inpatients with depression, but found no relationship between anxiety symptoms and impairment in general cognitive function as measured by the Mini Mental Status Examination (MMSE) and the Clock Drawing Test at admission to hospital (Bendixen et al., 2018). The group did however not include other measures of cognition.

According to attentional control theory (Eysenck and Derakshan, 2011; Eysenck et al., 2007), anxiety leads to enhanced focus on threat information and leaves fewer resources available for task relevant stimuli. Consequently, anxiety can result in domain specific dysfunction, such as problems with tasks involving inhibition of irrelevant information and attention switching. In line with attentional control theory, studies with healthy, community-dwelling older adults have indicated that subclinical symptoms of anxiety are related to poorer performance in specific cognitive domains. Impairments have been reported particularly in relation to executive functions, such as processing speed/shifting attention and inhibition (Beaudreau and O'hara, 2009; Yochim et al., 2013), but also in episodic memory (Stillman et al., 2012; Yochim et al., 2013). Similarly, in younger adults, major depressive disorder (MDD) with a comorbid anxiety disorder has been linked to greater executive dysfunction and psychomotor slowing compared to MDD alone (Basso et al., 2007), particularly in switching attention and inhibition functioning (Lyche et al., 2011).

More research is needed to clarify the potentially complex relationship between anxiety symptoms, depression, and cognitive function in late life. LLD is a heterogeneous disorder in which some individuals are assumed to experience a reduction in cognitive abilities over time, so it is important to clarify whether anxiety symptoms are a contributing factor to this decline. The overall aim of the present study was therefore to investigate the impact of comorbid anxiety symptom severity on cognitive function across several domains in patients admitted for in-hospital treatment for LLD. A broad set of cognitive tasks were included to measure performance in several cognitive domains, such as general cognitive function measured by the MMSE, different components of episodic memory, word fluency, and measures of executive functions including processing speed and attention switching. Anxiety was measured by the anxiety subscale of the Hospital Anxiety and Depression Scale (HADS-A) at admission to hospital. The objectives of the current study were (1) to assess the impact of anxiety symptom severity on change in performance across the cognitive domains during the hospital stay; and (2) to analyze anxiety symptom severity and how it affects cognitive performance at admission and at discharge from hospital. Finally using a cutoff on the HADS-A of ≥8 in exploratory analyses (3), we compare performance on the cognitive tasks between patients above and below the cutoff for clinically significant anxiety symptoms, both a) in relation to change in cognitive performance between admission and discharge, and b) cognitive performance at admission and at discharge.

We hypothesized that there would be an overall additive effect of comorbid anxiety symptoms in LLD on dysfunction across the cognitive domains. Although the literature on comorbid anxiety symptoms in depression and cognitive function is scarce, we reasoned that specific domains including executive functioning and episodic memory would be more affected by co-occurring anxiety than other cognitive domains. To our knowledge, this is the first study to examine coexisting anxiety symptoms and their associations with functioning across several cognitive domains during hospital treatment for LLD.

## 2 Methods

### 2.1 Design

We used data from the Prognosis of Depression in the Elderly (PRODE) sample. PRODE is a Norwegian multicenter, observational, prospective study of older inpatients treated for depression in nine departments of old-age psychiatry, previously described in Borza et al. (2015).

### 2.2 Patients

Persons were eligible for inclusion in the PRODE study if they were 60 years or older referred to specialist health care service for treatment of depression, not successfully treated in primary health care. For detailed information see Borza et al. (2015). Patients with dementia who had severe aphasia and patients with life-threatening diseases were not included. The participating patients and caregivers were given oral and written information about the study, and they subsequently gave written consent to participate. For patients without the capacity to give written consent, their next of kin gave consent on behalf of the patient. The study was approved by the Regional Committee of Medical Research Ethics and the Privacy and Data Protection Officer at Oslo University Hospital.

A total of 169 patients from nine centers were included in the PRODE sample between December 2009 and January 2013. Previous analyses showed no difference in age and sex between those who agreed and those who refused to participate (Borza et al., 2015). Nine patients were excluded because they were outpatients, fourteen patients were excluded for having been diagnosed with dementia during the hospital stay, and four patients were excluded because of missing data on anxiety level at admission. The current study ultimately included data for 142 older adult inpatients with depression.

### 2.3 Measurements

#### 2.3.1 Anxiety and depression scales

Anxiety and depression symptoms at admission and discharge from hospital were measured using the Norwegian version of the HADS (Zigmond and Snaith, 1983). The scale consists of 14 items, where seven items assess anxiety symptoms (HADS-A, e.g. “I feel tense or wound up”), and seven items address depressions symptoms (HADS-D, e.g. “I have lost interest in my appearance”). The items are rated on a 4-point scale ranging from 0 to 3. Higher score on HADS-A and HADS-D indicates more severe anxiety and depression, respectively. The scale has been validated in the Norwegian language, and the internal consistency reliability score (Cronbachs's alpha) has been found to vary between 0.77 and 0.88 for HADS-A, and between 0.70 and 0.88 for the HADS-D (Leiknes et al., 2016). The scale has proved to be a reliable and valid screening tool of severity and caseness of anxiety and depression in a variety of different samples (Helvik et al., 2011). To identify patients with clinical significant anxiety symptoms, we used the most common cutoff (≥8) on the anxiety subscale of the Hospital Anxiety and Depression Scale (HADS-A) and divided patients into groups indicating anxiety versus no anxiety (Bjelland et al., 2002).

#### 2.3.2 Cognitive measures

Cognitive assessment was done at admission and discharge from hospital. *General cognitive function* was measured using the Norwegian revised version of the Mini Mental Status Examination (MMSE-NR) (Folstein et al., 1975; Strobel and Engedal, 2008). The scale includes 20 simple questions and tasks that measures orientation, memory, arithmetic skills, language and basic motor abilities. Scores range from 0 to 30 and a higher score indicates better overall cognitive function. The scale has acceptable test-retest reliability (≥0.7) (Strobel and Engedal, 2008).

Word fluency was measured by two subtests of the Controlled Oral Word Association Test (COWAT). *Letter fluency* is measured as the total number of words the patient is able to produce starting with the letters F, A and S within a time limit of 1 min for each letter. Similarly, *category fluency* is measured as the total number of items named for the two categories “animal” and “clothing” (Benton, 1967). Acceptable test-retest reliability (0.74) has been proven for letter fluency (Ruff et al., 1996).

Episodic memory was measured by three subtasks of the Ten Word Test (Consortium to Establish a Registry for Alzheimer's Disease, CERAD) (Morris et al., 1988). The test consists of ten words presented and learned across three trials. *Immediate recall* is measured as the number of words the subject is able to recall across the three trials, with a total possible score of 30. *Delayed recall* is measured as the number of words the subject is able to reproduce after a delay of 10 min. Subjects are then given a list of ten novel words mixed with the ten words from the original list. *Recognition* is measured as the total number of correct positive and negative responses of whether each word was part of the original list or not, with a total possible score of 20. Test-retest reliability scores for the three subtasks are shown to range between 0.5-0.8 (Welsh-Bohmer and Mohs, 1997).

*Processing speed and attention switching (executive function)* were measured using two subtests of the Trail Making Test (TMT), TMT-A and TMT-B (Reitan, 1958). In TMT-A patients are instructed to sequentially connect numbered dots as fast as possible. Time to complete the task is used as a measure of processing speed. In TMT-B the subject needs to alternate between number and letters and connect the dots in numerical and alphabetical order as fast as possible. Time to complete the task is used as a measure of processing speed and attention switching. Results on the TMT were scored according to existing age-adjusted norms derived from Ivnik et al. (1996). Test-retest reliability is proven to be acceptable for TMT-A and good for TMT-B (0.75 and 0.85, respectively) (Giovagnoli et al., 1996).

#### 2.3.3 Demographic and clinical characteristics

Information on demographic characteristics and psychiatric history, including previous depressive episodes and age of onset of the first lifetime depressive episode, was obtained from case notes and structured interviews with patients and caregivers at admission. Diagnoses were established during hospital stay according to ICD-10 criteria (World Health Organization (WHO), 1993). Medications were classified according to the Anatomical and Therapeutic Chemical classification system. Use of psychotropic medications at admission and discharge was defined as the number of antidepressants, anxiolytics, hypnotics, antipsychotics, antidementia drugs, lithium, antiepileptics, and antiparkinsonian drugs patients were using at admission and discharge from hospital. Physical health was measured by the General Medical Health Rating (GMHR), a one-item scale with four categories (excellent, good, fair, and poor). GMHR was dichotomized into good (excellent/good) and poor (fair/poor) health status. High interrater reliability is reported for GMHR (weighted kappa = 0.91) (Lyketsos et al., 1999). Marital status was dichotomized into single (including singles, divorced or separated patients, and widows/widowers) and not single (married or living together with a partner).

### 2.4 Procedure

Standardized measures were administered by health professionals at admission and at discharge. Health professionals working in the involved departments received training in the standardized administration procedure before the start of the study and twice a year during the study period. Evaluation of eligibility of patients and inclusion in the study was done as soon as possible after admission to hospital by the trained health professionals. The mean number of days from admission to inclusion was 5.6 days (standard deviation (SD) = 6.0).

There was no treatment protocol; treatment varied across patients and study centers and included a range of different approaches. All patients received multidisciplinary treatment, combining medications and various therapeutic approaches. Among the patients, 90.1% received psychotropic medications at hospital admission and 94.4% received psychotropic medications at discharge. A total of 39 patients (27.5%) received Electroconvulsive therapy during the hospital stay, with an average of 12.7 (SD = 6.0) treatments. Discharge from hospital was based on the clinical procedure at each department, and the discharge assessment was done as close as possible to the discharge date.

### 2.5 Statistical analysis

All statistical analyses were conducted using the Statistical Program for Social Science Package (SPSS v. 25.0) and Statistical Analysis System (SAS v. 9.4). Imputation for MMSE-NR and HADS was performed for cases with 50% or fewer missing values on the scale. The empirical distribution for each item on the scale was determined, and a random number drawn from that distribution was used to replace the missing value. In the current sample, three values at admission and one at discharge for items on the MMSE-NR scale and one value at admission for an item on the HADS-A scale were imputed.

Patient characteristics were presented as means and SDs or frequencies and percentages, as appropriate. The HADS-A admission score was used in primary analyses. For exploratory analyses, the HADS-A admission score was dichotomized into two groups with a cutoff score of ≥8 for caseness of anxiety. Patients in different anxiety groups were compared using independent samples t-test and χ^2^-test.

Because patients were included from different centers, data could exhibit a hierarchical structure, while repeated measurements for patients imply within-patient correlations. To correctly adjust all estimates for within-patient and within-center correlations, random effects for patients nested within the centers were entered in all proceeding models. Center-level was eliminated if negligible or not present.

Six linear mixed models, one for each cognitive test measured as a continuous variable, were estimated using the SAS MIXED procedure. Time between admission and discharge, the HADS-A admission score, and the interaction between HADS-A and time were entered as fixed effects. For categorical tests, TMT-A, and TMT-B, generalized linear mixed models with the same fixed effects were estimated (SAS GLIMMIX procedure). A significant interaction term would imply that there are overall differences in association between HADS-A admission score and cognitive test at admission and discharge. In post hoc analysis, the models were explored further and the associations at each time point and differences between time points for varying HADS-A values were quantified. HADS-A score was substituted with a dichotomized HADS-A in exploratory analyses.

All regression models were adjusted for depression severity at admission (HADS-D), previous depressive episodes, and number of psychotropic medications across admission and discharge in addition to sex, age, and education. Because TMT-A and TMT-B were scored according to age-based norms, only adjustment for depression severity at admission, sex and education was performed. The cognitive test scores are highly correlated, so we implemented no adjustment for multiple testing. P-values are reported as they are in all models, and significance level was set to the conventional 5% in all analyses.

## 3 Results

### 3.1 Descriptive findings

The mean HADS-A score at admission was 11.4 (SD = 4.7) and decreased significantly to 6.5 (SD = 4.5) at discharge (*p* < 0.001), while the mean HADS-D score decreased significantly from 11.9 (SD = 4.8) at admission to 6.4 (SD = 4.5) at discharge (*p* < 0.001). Demographic and clinical characteristics at admission are shown in Table 1.

**Table 1:** Demographic and clinical characteristics^a^.

	Value
Age, mean (SD)	76.3 (6.8)
Women, n (%)	105 (73.9)
Marital status, single, n (%)	84 (59.2)
Years of education, mean (SD) (*n* = 135)	10.0 (3.0)
Days of stay, mean (SD)	68.5 (47.0)
Number of psychotropic medications at admission, mean (SD)	2.1 (1.4)
Number of psychotropic medications at discharge, mean (SD)	2.3 (1.2)
GMHR, Good, ​n (%)	73 (51.4)
Age at onset of the first lifetime depressive episode, <60 years, n (%) (*n* = 135)	65 (48.1)
Duration of depressive episode, <13 weeks, n (%) (*n* = 139)	62 (44.6)
Previous depressive episode, n (%) (*n* = 141)	97 (68.8)
Recurrent depression (F33, ICD-10), n (%)	83 (58.5)
Bipolar diagnosis (F31, ICD-10), n (%)	9 (6.3)
Depression with psychosis (F32.3/F33.3, ICD-10), n (%)	17 (12.0)
Patients with personality disorder (F60–F69, ICD-10), n (%)	2 (1.4)
HADS-A at admission, mean (SD)	11.4 (4.7)
HADS-A at discharge, mean (SD) (*n* = 130)	6.5 (4.5)
HADS-D at admission, mean (SD)	11.9 (4.8)
HADS-D at discharge, mean (SD) (*n* = 130)	6.4 (4.5)

*Abbreviations*: SD = standard deviation; GMHR = General Medical Health Rating; HADS-A = Anxiety subscale of Hospital Anxiety and Depression Scale; HADS-D = Depression subscale of Hospital Anxiety and Depression Scale; ICD = International Statistical Classification of Diseases and Related Health Problems

a All values are from the time of admission and with *n* = 142 if not otherwise specified.

Demographic and clinical characteristics across dichotomized HADS-A groups based on cutoff score (anxiety versus no anxiety) are given in Table 2. Patients above cutoff on HADS-A scored significantly higher on HADS-A at discharge, HADS-D at admission, stayed longer in the hospital, and used more psychotropic medications at admission than patients below the cutoff. Age, sex, education, marital status, psychotropic medications at discharge, and GMHR did not differ across HADS-A groups. There was no difference in distribution of diagnoses (recurrent depression, bipolar disorder, depression with psychosis, personality disorder) or age of onset of first depression episode, duration of depressive episode or occurrence of previous depressive episodes across HADS-A groups.

**Table 2:** Demographic and clinical characteristics across anxiety groups^a^.

	HADS-A groups		
	Anxiety (HADS-A≥8) (*n* = 112)	No anxiety (HADS-A<8) (*n* = 30)	*p*
Age, mean (SD)	76.2 (6.7)	76.8 (7.6)	0.663
Women, n (%)	85 (75.9)	20 (66.7)	0.307
Marital status, Single, n (%)	48 (42.9)	10 (33.3)	0.346
Years of education, mean (SD) (*n* = 135)	9.9 (2.9)	10.3 (3.2)	0.564
Days of stay in hospital, mean (SD)	72.7 (47.5)	52.7 (42.6)	**0.038**
Number of psychotropic medications at admission, mean (SD)	2.2 (1.4)	1.6 (1.2)	**0.039**
Number of psychotropic medications at discharge, mean (SD)	2.4 (1.2)	2.1 (1.3)	0.314
GMHR, Good, n (%)	56 (50.0)	17 (56.7)	0.516
HADS-A at discharge, mean (SD) (*n* = 130)	7.1 (4.5)	4.30 (3.8)	**0.004**
HADS-D at admission, mean (SD) (*n* = 132)	13.1 (4.1)	7.1 (4.4)	**<0.001**
HADS-D at discharge, mean (SD) (*n* = 130)	6.8 (4.5)	5.0 (4.3)	0.066
Age at onset of the first lifetime depressive episode, <60 years, n (%) (*n* = 139)	54 (50.5)	11 (39.3)	0.292
Duration of depressive episode, <13 weeks, n (%) (*n* = 139)	51 (46.8)	11 (36.7)	0.323
Previous depressive episode (s), n (%) (*n* = 141)	81 (72.3)	16 (55.2)	0.076
Recurrent depression (F33, ICD-10), n (%)	69 (61.6)	14 (46.7)	0.140
Bipolar diagnosis (F31, ICD-10), n (%)	7 (6.3)	2 (6.7)	0.934
Depression with psychosis (F32.3/F33.3, ICD-10), n (%)	13 (11.6)	4 (13.3)	0.796
Patients with personality disorder (F60–F69, ICD-10), n (%)	2 (1.8)	0 (0)	0.461

*Abbreviations:* SD = standard deviation; GMHR = General Medical Health Rating; HADS-A = Anxiety subscale of Hospital Anxiety and Depression Scale; HADS-D = Depression subscale of Hospital Anxiety and Depression Scale; ICD = International Statistical Classification of Diseases and Related Health Problems.

Values in bold denote statistical significance at the p < 0.05-level for main effects of time, anxiety and the interaction between time and anxiety.

a All values are from the time of admission and with n = 142 if not otherwise specified.

### 3.2 Anxiety and cognitive function

Table 3 displays the raw mean scores and SDs for each cognitive test at admission and discharge.

**Table 3:** Cognitive test scores at admission and discharge.

	Admission	Discharge
MMSE-NR	26.4 (3.1)	26.8 (3.1)
Immediate recall, mean (SD)	15.4 (5.1)	18.4 (5.2)
Delayed recall, mean (SD)	4.3 (2.4)	5.6 (2.4)
Recognition, mean (SD)	17.7 (2.5)	18.0 (2.4)
Word fluency, mean (SD)	28.8 (11.8)	31.8 (13.2)
Category fluency, mean (SD)	26.1 (9.0)	28.4 (10.1)
TMT-A		
Time better than 1 SD, n (%)	23 (19.7)	26 (23.4)
Time between 1 and 2 SD, n (%)	50 (42.7)	42 (37.8)
Time worse than 2 SD, n (%)	36 (30.8)	35 (31.5)
Not able to complete the test, n (%)	8 (6.8)	8 (7.2)
TMT-B		
Time better than 1 SD, n (%)	26 (22.6)	28 (24.8)
Time between 1 and 2 SD, n (%)	14 (12.2)	15 (13.3)
Time worse than 2 SD, n (%)	13 (11.3)	11 (9.7)
Not able to complete the test, n (%)	62 (53.9)	59 (52.2)

*Abbreviations*: SD = standard deviation, MMSE-NR = Mini Mental Status Examination, Norwegian revised version; TMT = Trail Making Test.

#### 3.2.1 Immediate recall

Overall, there were differences between time points regarding the association between HADS-A score and the immediate recall task (*p* = 0.037) with significantly higher immediate recall scores at discharge compared to admission for increasing HADS-A, but only for HADS-A values above 4 (Table 4). Exploratory analyses with the dichotomized HADS-A showed that there was no significant change in the immediate recall task score from admission to discharge in neither group, however overall the change was significantly different between the groups (*p* = 0.030 for interaction) (Table 4), where those with a score above cutoff for anxiety on HADS-A recalled significantly more words at discharge than at admission (*p* < 0.001); with no difference among those below cutoff.

**Table 4:** Results of linear mixed models for continuous cognitive test scores.

HADS-A continuous scale												
	MMSE-NR		Immediate recall		Delayed recall		Recognition		Word fluency		Category fluency	
	B(SE)	p	B(SE)	p	B(SE)	p	B(SE)	p	B(SE)	p	B(SE)	p
Time	0.47 (0.57)	0.404	0.78 (1.14)	0.496	1.81 (0.50)	**0.001**	0.12 (0.65)	0.847	4.80 (2.27)	**0.037**	2.01 (2.07)	0.334
HADS-A	0.17 (0.10)	0.081	−0.23 (0.18)	0.223	0.08 (0.08)	0.307	0.04 (0.10)	0.675	0.70 (0.41)	0.084	0.16 (0.33)	0.633
Time × HADS-A	−0.01 (0.05)	0.781	0.20 (0.09)	**0.037**	−0.04 (0.04)	0.340	0.01 (0.05)	0.783	−0.15 (0.19)	0.438	0.03 (0.17)	0.866
Sex, man	0.67 (0.54)	0.218	−1.21 (0.99)	0.226	−1.05 (0.43)	0.017	−0.29 (0.46)	0.526	−5.68 (2.40)	0.020	−5.11 (1.72)	0.004
Age	−0.07 (0.04)	0.048	−0.11 (0.07)	0.123	−0.07 (0.03)	0.024	−0.05 (0.03)	0.110	−0.05 (0.17)	0.762	−0.20 (0.12)	0.095
Education	0.31 (0.08)	<0.001	0.37 (0.15)	0.016	0.22 (0.07)	0.001	0.05 (0.07)	0.512	1.52 (0.36)	<0.001	1.14 (0.26)	<0.001
HADS-D	−0.17 (0.07)	0.014	0.05 (0.13)	0.693	0.05 (0.05)	0.410	0.001 (0.06)	0.980	−0.56 (0.30)	0.064	−0.19 (0.22)	0.380
Psychotropic medications	−0.03 (0.12)	0.825	−0.47 (0.25)	0.058	−0.09 (0.11)	0.406	0.01 (0.13)	0.927	0.75 (0.51)	0.145	−0.34 (0.43)	0.423
Previous depressive episode, yes	−0.01 (0.50)	0.979	0.04 (0.95)	0.966	−0.52 (0.43)	0.222	−0.52 (0.44)	0.237	−3.71 (2.32)	0.113	−4.11 (1.67)	0.015
HADS-A two groups												
Time	0.91 (0.45)	**0.046**	1.26 (0.91)	0.168	1.10 (0.40)	**0.007**	0.19 (0.51)	0.714	3.78 (1.75)	**0.033**	1.07 (1.59)	0.501
HADS-A≥8	2.17 (0.99)	**0.030**	−3.96 (1.95)	**0.044**	−0.10 (0.84)	0.904	0.40 (1.03)	0.699	3.32 (4.15)	0.439	−1.68 (3.36)	0.617
Time × HADS-A≥8	−0.73 (0.51)	0.154	2.25 (1.02)	**0.030**	0.33 (0.45)	0.463	0.16 (0.58)	0.791	−0.77 (1.98)	0.698	1.63 (1.79)	0.364
Sex, man	0.83 (0.54)	0.128	−1.16 (0.99)	0.242	−1.02 (0.43)	0.020	−0.21 (0.46)	0.639	−5.31 (2.42)	0.031	−4.95 (1.73)	0.005
Age	−0.08 (0.04)	0.039	−0.11 (0.07)	0.110	−0.07 (0.03)	0.023	−0.05 (0.03)	0.101	−0.06 (0.17)	0.714	−0.20 (0.12)	0.089
Education	0.31 (0.08)	<0.001	0.35 (0.15)	0.021	0.21 (0.07)	0.002	0.04 (0.07)	0.517	1.50 (0.37)	<0.001	1.13 (0.26)	<0.001
HADS-D	−0.12 (0.06)	0.044	0.12 (0.11)	0.282	0.04 (0.05)	0.366	0.01 (0.05)	0.795	−0.35 (0.27)	0.204	−0.10 (0.19)	0.614
Psychotropic medications	−0.05 (0.12)	0.660	−0.45 (0.25)	0.066	−0.09 (0.11)	0.436	0.02 (0.13)	0.909	0.74 (0.52)	0.153	−0.33 (0.43)	0.442
Previous depressive episode, yes	0.06 (0.51)	0.914	0.12 (0.95)	0.899	−0.53 (0.43)	0.220	−0.52 (0.44)	0.237	−3.56 (2.34)	0.131	−4.01 (1.67)	0.018

*Abbreviations*: B = regression coefficient; SE = standard error; MMSE-NR = Mini Mental Status Examination, Norwegian revised version; HADS-A = Anxiety subscale of Hospital Anxiety and Depression Scale; HADS-D = Depression subscale of Hospital Anxiety and Depression Scale.

Values in bold denote statistical significance at the p < 0.05-level for main effects of time, anxiety and the interaction between time and anxiety.

#### 3.2.2 General cognitive function

No association between HADS-A score and performance on MMSE was found. According to exploratory analyses with the dichotomized HADS-A, there was no overall difference in change in MMSE between those with anxiety below and above the cutoff. However, those with a score above cutoff for anxiety on HADS-A scored higher on MMSE at admission (*p* = 0.030) compared to those below cutoff.

#### 3.2.3 Delayed recall and word fluency

No association was found between HADS-A score and performance on the delayed recall task, or performance on the word category task. Patients overall did however remember more words at discharge compared to admission (*p* = 0.001) in the delayed recall task, and produced more words at discharge compared to admission in the word fluency tasks (*p* = 0.037) (Table 4). The same finding was present in exploratory analyses with dichotomized HADS-A as explanatory variable (Table 4).

#### 3.2.4 Recognition, category fluency, and processing speed/attention switching (executive function)

No association between continuous or dichotomized HADS-A score and performance on the recognition task, category fluency task (Table 4), TMT-A, or TMT-B (Table 5) was found. Neither were there any significant interactions present.

**Table 5:** Results of generalized linear mixed models for categorical cognitive test scores.

HADS-A continuous scale				
	TMT-A		TMT-B	
	B (SE)	p	B (SE)	p
Time	−0.31 (0.81)	0.692	0.15 (1.07)	0.891
HADS-A	−0.06 (0.13)	0.645	−0.14 (0.19)	0.485
Time × HADS-A	−0.008 (0.07)	0.911	−0.09 (0.09)	0.307
Sex, man	−0.02 (0.64)	0.975	−0.88 (1.16)	0.448
Education	−0.34 (0.10)	0.002	−0.81 (0.25)	0.002
HADS-D	0.11 (0.08)	0.195	0.15 (0.15)	0.334
Psychotropic medications	0.31 (0.17)	0.076	0.45 (0.27)	0.104
Previous depressive episode, yes	0.21 (0.63)	0.746	−0.10 (1.14)	0.932
HADS-A 2 groups				
Time	−0.30 (0.66)	0.653	−0.73 (0.83)	0.416
HADS-A≥8	−0.49 (1.34)	0.715	−1.20 (2.02)	0.553
Time × HADS-A≥8	−0.14 (0.74)	0.846	−0.20 (0.99)	0.840
Sex, man	−0.08 (0.64)	0.905	−1.18 (1.13)	0.298
Education	−0.34 (0.10)	0.001	−0.78 (0.24)	0.001
HADS-D	0.10 (0.07)	0.198	0.04 (0.13)	0.780
Psychotropic medications	0.31 (0.17)	0.072	0.44 (0.26)	0.094
Previous depressive episode, yes	0.20 (0.63)	0.748	−0.07 (1.10)	0.946

*Abbreviations*: B = regression coefficient; SE = standard error; HADS-A = Anxiety subscale of Hospital Anxiety and Depression Scale; HADS-D = Depression subscale of Hospital Anxiety and Depression Scale; TMT = Trail Making Test.

## 4 Discussion

This study examined the relationship between anxiety symptoms at admission of hospitalization and change in cognitive function across treatment of LLD, and between anxiety symptoms at admission and cognitive function at hospital admission and discharge. To our knowledge, no studies have looked at coexisting anxiety symptoms measured by HADS-A and their associations with cognitive function in several cognitive domains in older persons with clinical depression. Higher level of comorbid anxiety symptoms at admission was not associated with reduced cognitive function in any of the cognitive domains in patients treated for depression in this study. The findings are therefore in line with the literature suggesting that anxiety does not lead to an increased risk of cognitive dysfunction (de Bruijn et al., 2014). Based on previous findings, we reasoned that episodic memory would be particularly negatively affected by comorbid anxiety symptoms. Although patients with more pronounced anxiety symptoms at admission scored significantly higher on the immediate recall task at discharge compared to admission, there was no association between anxiety symptom level and the immediate recall task itself. Similarly, there was no association between anxiety symptom level and performance on the delayed recall and recognition tasks. The findings are therefore in contrast to those of DeLuca et al. (2005), where having a comorbid anxiety disorder with depression was associated with an accelerated memory decline relative to only having a diagnosis of depression. Our findings suggest that symptoms of anxiety that occur together with a diagnosis of depression do not lead to a greater reduction in memory during hospital stay. There was no association between anxiety symptom severity or anxiety groups and cognitive function neither at admission nor at discharge, except for performance on the MMSE at admission, where it was found that those above cutoff for anxiety scored higher compared to those without anxiety. Anxiety has been proposed in some circumstances to be beneficial for cognitive performance. In a series of studies by Bierman and colleagues (Bierman et al., 2005; Bierman et al., 2008), mild anxiety in community-dwelling older people, as measured by the HADS-A, was related to better performance, while severe anxiety was negatively associated with performance. Others have also posited that state anxiety does not need to be detrimental but rather could be favorable for cognition when controlling for confounders (Potvin et al., 2013). As there was no difference in general cognitive function in our sample at discharge from hospital, our findings are also in line with the study of Bendixen et al. (2019), where it was found that initial anxiety among older adults in specialist mental health services did not predict future decline in general cognitive function as measured by MMSE. Throughout hospital stay there was a significant improvement in number of words remembered on the delayed recall task, and in number of words produced in the word fluency task. Initial problems in cognitive function related to depression and/or anxiety at admission were most likely present among patients, and improvements might have been caused by treatment and thus reductions in psychopathological severity. Patients with depression improve in cognitive function during antidepressant treatment (Butters et al., 2000; Yoo et al., 2015), although they are still more impaired than older persons without psychiatric illness after treatment (Butters et al., 2000). Alternatively, some of the improvement might have resulted from practice effects. For instance, older men with and without impairments in delayed memory function at baseline showed practice effects over time, while the beneficial effects of practice disappeared after five years for those with impairments at baseline (Mathews et al., 2013). A substantial number of the patients in the current sample experienced a significant amount of anxiety at admission to hospital. Nearly 80% of the patients scored above the cutoff for clinical significant anxiety symptoms. Although comorbid anxiety symptoms were not associated with additional cognitive problems in our sample of patients in treatment for late-life depression, we found that patients above the cutoff for anxiety at admission also seemed to need longer treatment time, used more medications and had higher anxiety at discharge than patients below the cutoff score. The findings indicate that patients with late-life depression and comorbid anxiety symptoms have more severe illness than those without anxiety, consistent with studies that have linked comorbid anxiety to worse treatment response (Andreescu et al., 2007), and more severe depression (Bendixen et al., 2018; Lenze et al., 2000). Thus, it is important to target and treat anxiety in patients with late-life depression.

### 4.1 Limitations and strengths

Cognitive test scores are correlated, and the Bonferroni correction is overly conservative in such cases, and lowers the chance of detecting real differences (Type 2 error). As we hope that our findings encourage future studies, replication and further exploration of the association between comorbid anxiety symptoms in late-life depression and cognition, p-values were reported without adjustment for multiple testing. The results should therefore be interpreted with caution. Our main aim was to study the effect of comorbid anxiety symptoms on cognitive function among patients with depression, and a control group was not considered necessary. Based on previous research on depression, patients were most likely cognitively impaired compared to the healthy population (Koenig et al., 2014; Morimoto and Alexopoulos, 2013). As the study did not include any control group, it is not possible to compare direct effects of depression on cognitive function, and we are only able to make assumptions based on the established literature. The study's strengths were the use of well-established and validated assessment scales, inclusion of several cognitive tasks, a representative sample of the clinical population, and robust statistical methods. Because few exclusion criteria were used and because of the observational and prospective design, the sample is representative of everyday clinical practice in psychiatric specialist health care for older adults in Norway.

### 4.2 Future directions

Although we did not find any association between anxiety symptoms and cognitive dysfunction in our sample of inpatients treated for LLD, it might be that comorbid anxiety influence cognition over a longer time-period. It has been suggested that anxiety has a moderate effect over short time periods, which increases when followed up over a longer period (Petkus et al., 2017). Previous findings show that anxiety is tied to greater memory decline over 4 years (DeLuca et al., 2005) and is associated with a genetic risk for dementia (Petkus et al., 2017). Others, however, have found that neither anxiety disorders nor anxiety symptoms as measured by HADS-A were associated with increased risk for developing dementia (de Bruijn et al., 2014). Future studies should therefore investigate anxiety symptoms in depressed patients over a longer time period after treatment. Moreover, our results confirm the findings of Bendixen and Engedal (2016), where anxiety symptoms seem to be common among patients with LLD. As these symptoms occur to be persistent (Bendixen et al., 2019), future work should investigate whether specifically treating anxiety symptoms in depressed patients lead to better treatment outcomes.

## 5 Conclusion

There was no additive effect of comorbid anxiety symptoms on cognitive dysfunction in late-life depression in our sample of inpatients.

## Declarations

### Author contribution statement

Liva Jenny Martinussen, Ina Selseth Almdahl, Maria Stylianou Korsnes: Analyzed and interpreted the data; Wrote the paper.

Jūratė Šaltytė Benth: Analyzed and interpreted the data; Contributed reagents, materials, analysis tools or data; Wrote the paper.

Tom Borza, Geir Selbaek: Conceived and designed the experiments; Performed the experiments; Wrote the paper.

Bodil Mcpherson: Performed the experiments; Wrote the paper.

### Funding statement

The study reported in this article was supported by the Old Age Psychiatry Research Group, Oslo University Hospital, Norway. The original study was supported by unrestricted grants from the South-Eastern Norway Regional Health Authority (grant number: 2010088) and Innlandet Hospital Trust, Norway (grant number: 150201). These institutions had no further role in study design; in the collection, analysis, and interpretation of data; in the writing of the report; or in the decision to submit the paper for publication.

### Competing interest statement

The authors declare no conflict of interest.

### Additional information

The observational study described in this paper was registered at ClinicalTrials.gov under the registration number NCT01952366.

## References

[bib1] Andreescu C., Lenze E.J., Dew M.A., Begley A.E., Mulsant B.H., Dombrovski A.Y. (2007). Effect of comorbid anxiety on treatment response and relapse risk in late-life depression: controlled study. Br. J. Psychiatry.

[bib2] Basso M.R., Lowery N., Ghormley C., Combs D., Purdie R., Neel J. (2007). Comorbid anxiety corresponds with neuropsychological dysfunction in unipolar depression. Cogn. Neuropsychiatry.

[bib3] Beaudreau S.A., O'hara R. (2009). The association of anxiety and depressive symptoms with cognitive performance in community-dwelling older adults. Psychol. Aging.

[bib4] Bendixen A.B., Engedal K. (2016). Anxiety among older psychiatric patients: a hidden comorbidity?. Aging Ment. Health.

[bib5] Bendixen A.B., Engedal K., Selbæk G., Benth J.Š., Hartberg C.B. (2019). Anxiety symptom levels are persistent in older adults with a mental disorder: a 33-month follow-up study. Int. J. Geriatr. Psychiatry.

[bib6] Bendixen A.B., Engedal K., Selbæk G., Hartberg C.B. (2018). Anxiety symptoms in older adults with depression are associated with suicidality. Dement. Geriatr. Cognit. Disord..

[bib7] Benton A.L. (1967). Problems of test construction in the field of aphasia. Cortex.

[bib8] Bierman E.J.M., Comijs H.C., Jonker C., Beekman A.T.F. (2005). Effects of anxiety versus depression on cognition in later life. Am. J. Geriatr. Psychiatry.

[bib9] Bierman E.J.M., Comijs H.C., Rijmen F., Jonker C., Beekman A.T. (2008). Anxiety symptoms and cognitive performance in later life: results from the longitudinal aging study Amsterdam. Aging Ment. Health.

[bib10] Bjelland I., Dahl A.A., Haug T.T., Neckelmann D. (2002). The validity of the Hospital Anxiety and Depression Scale: an updated literature review. J. Psychosom. Res..

[bib11] Borza T., Engedal K., Bergh S., Benth J.Š., Selbaek G. (2015). The course of depression in late life as measured by the Montgomery and Asberg Depression Rating Scale in an observational study of hospitalized patients. BMC Psychiatry.

[bib12] Butters M.A., Becker J.T., Nebes R.D., Zmuda M.D., Mulsant B.H., Pollock B.G., Reynolds C.F. (2000). Changes in cognitive functioning following treatment of late-life depression. Am. J. Psychiatry.

[bib13] de Bruijn R.F., Direk N., Mirza S.S., Hofman A., Koudstaal P.J., Tiemeier H., Ikram M.A. (2014). Anxiety is not associated with the risk of dementia or cognitive decline: the Rotterdam Study. Am. J. Geriatr. Psychiatry.

[bib14] DeLuca A.K., Lenze E.J., Mulsant B.H., Butters M.A., Karp J.F., Dew M.A. (2005). Comorbid anxiety disorder in late life depression: association with memory decline over four years. Int. J. Geriatr. Psychiatry.

[bib15] Diniz B.S., Butters M.A., Albert S.M., Dew M.A., Reynolds C.F. (2013). Late-life depression and risk of vascular dementia and Alzheimer's disease: systematic review and meta-analysis of community-based cohort studies. Br. J. Psychiatry.

[bib16] Eysenck M.W., Derakshan N. (2011). New perspectives in attentional control theory. Personal. Individ. Differ..

[bib17] Eysenck M.W., Derakshan N., Santos R., Calvo M.G. (2007). Anxiety and cognitive performance: attentional control theory. Emotion.

[bib18] Folstein M.F., Folstein S.E., McHugh P.R. (1975). “Mini-mental state”: a practical method for grading the cognitive state of patients for the clinician. J. Psychiatr. Res..

[bib19] Giovagnoli A.R., Del Pesce M., Mascheroni S., Simoncelli M., Laiacona M., Capitani E. (1996). Trail making test: normative values from 287 normal adult controls. Ital. J. Neurol. Sci..

[bib20] Gulpers B., Ramakers I., Hamel R., Köhler S., Voshaar R.O., Verhey F. (2016). Anxiety as a predictor for cognitive decline and dementia: a systematic review and meta-analysis. Am. J. Geriatr. Psychiatry.

[bib21] Helvik A.-S., Engedal K., Skancke R.H., Selbaek G. (2011). A psychometric evaluation of the Hospital Anxiety and Depression Scale for the medically hospitalized elderly. Nord. J. Psychiatry.

[bib22] Ivnik R.J., Malec J.F., Smith G.E., Tangalos E.G., Petersen R.C. (1996). Neuropsychological tests' norms above age 55: COWAT, BNT, MAE token, WRAT-R reading, AMNART, STROOP, TMT, and JLO. Clin. Neuropsychol..

[bib23] Koenig A.M., Bhalla R.K., Butters M.A. (2014). Cognitive functioning and late-life depression. J. Int. Neuropsychol. Soc..

[bib24] Leiknes K.A., Dalsbø T.K., Siqveland J. (2016). Psychometric Assessment of the Norwegian Version of the Hospital Anxiety and Depression Scale (HADS).

[bib25] Lenze E.J., Mulsant B.H., Shear M.K., Schulberg H.C., Dew M.A., Begley A.E. (2000). Comorbid anxiety disorders in depressed elderly patients. Am. J. Psychiatry.

[bib26] Lyche P., Jonassen R., Stiles T., Ulleberg P., Landrø N. (2011). Attentional functions in major depressive disorders with and without comorbid anxiety. Arch. Clin. Neuropsychol..

[bib27] Lyketsos C.G., Galik E., Steele C., Steinberg M., Rosenblatt A., Warren A. (1999). The General Medical Health Rating: a bedside global rating of medical comorbidity in patients with dementia. J. Am. Geriatr. Soc..

[bib28] Löwe B., Spitzer R.L., Williams J.B., Mussell M., Schellberg D., Kroenke K. (2008). Depression, anxiety and somatization in primary care: syndrome overlap and functional impairment. Gen. Hosp. Psychiatry.

[bib29] Mathews M., Abner E., Caban-Holt A., Kryscio R., Schmitt F. (2013). CERAD practice effects and attrition bias in a dementia prevention trial. Int. Psychogeriatr..

[bib30] Morimoto S.S., Alexopoulos G.S. (2013). Cognitive deficits in geriatric depression: clinical correlates and implications for current and future treatment. Psychiatr. Clin. N. Am..

[bib31] Morris J.C., Mohs R., Rogers H., Fillenbaum G., Heyman A. (1988). Consortium to establish a registry for Alzheimer's disease (CERAD) clinical and neuropsychological assessment of Alzheimer's disease. Psychopharmacol. Bull..

[bib32] Petkus A.J., Reynolds C.A., Wetherell J.L., Kremen W.S., Gatz M. (2017). Temporal dynamics of cognitive performance and anxiety across older adulthood. Psychol. Aging.

[bib33] Potvin O., Bergua V., Meillon C., Le Goff M., Bouisson J., Dartigues J.-F., Amieva H. (2013). State anxiety and cognitive functioning in older adults. Am. J. Geriatr. Psychiatry.

[bib34] Reitan R.M. (1958). Validity of the Trail Making Test as an indicator of organic brain damage. Percept. Mot. Skills.

[bib35] Ruff R., Light R., Parker S., Levin H. (1996). Benton controlled oral word association test: reliability and updated norms. Arch. Clin. Neuropsychol..

[bib36] Stillman A.N., Rowe K.C., Arndt S., Moser D.J. (2012). Anxious symptoms and cognitive function in non-demented older adults: an inverse relationship. Int. J. Geriatr. Psychiatry.

[bib37] Strobel C., Engedal K. (2008). MMSE-NR. Norwegian Revised Mini Mental Status Evaluation. Revised and Expanded Manual.

[bib38] Welsh-Bohmer K.A., Mohs R.C. (1997). Neuropsychological assessment of Alzheimer's disease. Neurology.

[bib39] World Health Organization (WHO) (1993). The ICD-10 Classification of Mental and Behavioural Disorders: Diagnostic Criteria for Research.

[bib40] Yochim B.P., Mueller A.E., Segal D.L. (2013). Late life anxiety is associated with decreased memory and executive functioning in community dwelling older adults. J. Anxiety Disord..

[bib41] Yoo I., Woo J.-M., Lee S.-H., Fava M., Mischoulon D., Papakostas G.I. (2015). Influence of anxiety symptoms on improvement of neurocognitive functions in patients with major depressive disorder: a 12-week, multicenter, randomized trial of tianeptine versus escitalopram, the CAMPION study. J. Affect. Disord..

[bib42] Zigmond A.S., Snaith R.P. (1983). The hospital anxiety and depression scale. Acta Psychiatr. Scand..

